# CXCL4 Contributes to the Pathogenesis of Chronic Liver Allograft Dysfunction

**DOI:** 10.1155/2016/9276986

**Published:** 2016-12-08

**Authors:** Jing Li, Bin Liu, Yuan Shi, Ke-Liang Xie, Hai-Fang Yin, Lu-nan Yan, Wan-yee Lau, Guo-Lin Wang

**Affiliations:** ^1^Department of Anesthesiology, General Hospital of Tianjin Medical University, Tianjin 300052, China; ^2^Department of Critical Care Medicine, General Hospital of Tianjin Medical University, Tianjin 300052, China; ^3^Organ Transplantation Center, Tianjin First Center Hospital, Nankai District, Tianjin 300192, China; ^4^Department of Cell Biology and Research Center of Basic Medical Science, Tianjin Medical University, Qixiangtai Road, Heping District, Tianjin 300070, China; ^5^Liver Transplantation Center, West China Hospital of Sichuan University, Chengdu 610041, Sichuan Province, China; ^6^Faculty of Medicine, The Chinese University of Hong Kong, Prince of Wales Hospital, Sha Tin, Hong Kong

## Abstract

Chronic liver allograft dysfunction (CLAD) remains the most common cause of patient morbidity and allograft loss in liver transplant patients. However, the pathogenesis of CLAD has not been completely elucidated. By establishing rat CLAD models, in this study, we identified the informative CLAD-associated genes using isobaric tags for relative and absolute quantification (iTRAQ) proteomics analysis and validated these results in recipient rat liver allografts. CXCL4, CXCR3, EGFR, JAK2, STAT3, and Collagen IV were associated with CLAD pathogenesis. We validated that CXCL4 is upstream of these informative genes in the isolated hepatic stellate cells (HSC). Blocking CXCL4 protects against CLAD by reducing liver fibrosis. Therefore, our results indicated that therapeutic approaches that neutralize CXCL4, a newly identified target of fibrosis, may represent a novel strategy for preventing and treating CLAD after liver transplantation.

## 1. Introduction

Despite ongoing advances in organ preservation and immunosuppression therapy, chronic liver allograft dysfunction (CLAD) remains the most common cause of patient morbidity and allograft loss in liver transplant patients [1]. Liver allograft biopsy studies have shown that 37% of recipients who survive longer than 5 years present with CLAD, which adversely impacts the long-term allograft survival [2]. The morphologic hallmarks of CLAD include hepatocyte necrosis, bile duct damage or disappearance, hepatic obliterative arteriopathy, and liver fibrosis [3, 4]. Liver graft fibrosis in particular is a major determinant of clinical outcomes in CLAD patients [5]. These histopathologic changes associated with CLAD can be attributed to immunological and nonimmunological factors, including ischemia/reperfusion (I/R) injury, acute or chronic rejection, drug toxicity, and de novo or recurrent disease [6, 7]. Development of novel strategies that prevent CLAD-associated damage is the key to ultimate graft survival. Emerging evidence has indicated that chemokines and their receptors are associated with the development of CLAD [2, 8]. CXCL4 is secreted by platelets that specifically activate the CXCR3 receptor, which is involved in the control of many biological processes, including hematopoiesis, angiogenesis, fibrogenesis, and innate and acquired immune responses [1]. CXCL4 expression has been observed in liver allografts throughout all stages of transplantation [9], indicating that CXCL4 and its receptor CXCR3 have important roles in the pathogenesis of CLAD after liver transplantation [10]. However, its role in the pathogenesis of CLAD has not been completely elucidated. In this study, we applied isobaric tags for relative and absolute quantification (iTRAQ) proteomics analysis to identify that CXCL4 is an informative gene in CLAD. We showed that the severity of CLAD was significantly ameliorated after CXCL4 neutralization by monoclonal antibody (CXCL4mab) treatment in a rat model of CLAD.

## 2. Materials and Methods

### 2.1. Serum Samples and Liver Biopsies from Patients with CLAD

CXCL4 serum concentrations were determined in 93 liver transplant patients with CLAD and 20 healthy subjects. After histopathological evaluation, we extracted total hepatic mRNA from paraffin-embedded liver biopsies of the patients with CLAD and 30 patients without CLAD. CXCL4 serum concentrations were determined by enzyme-linked immunosorbent assay (ELISA; R&D Systems, MN). Total mRNA from patients with and without CLAD was reverse-transcribed using SuperScript (Invitrogen). Quantitative reverse transcription polymerase chain reaction was carried out for CXCL4 with an Assay from Applied Biosystems (Hs00236998_m1). Target gene expression was normalized to 18S ribosomal RNA levels.

### 2.2. Rats and Establishment of Rat CLAD Models

Pathogen-free, healthy male BN (RT^1*n*^) and Lewis (RT^11^) rats weighing 250–300 g (purchased from the institute of Laboratory Animal Science, Chinese Academy of Medical Sciences) were used as liver donors and recipients, respectively. The animals were housed under standard conditions with 12-hour dark-light cycle and free access to water and food. Animal experiments were performed with the authorization of the Animal Care and Use Committee of the Tianjin Medical University. Isoflurane was administered as general inhalation anesthesia in all cases. All surgical procedures were performed under aseptic conditions by a single microsurgeon with the aid of a 4–40x magnification microscope (Leica Microsystems, Wetzlar, Germany). The two-cuff technique was used to establish rat orthotopic liver transplantations (OLT) models as previously described by Kamada and Calne [11]. Daily Cefazolin (40 mg/kg) was injected intramuscularly after the implantation operation for 5 days to prevent infection. Tacrolimus was donated by the Organ Transplantation Center of Tianjin First Center Hospital (Tianjin, China) and administered (0.1 mg/kg per day, injected intramuscularly) for 5 days after the implantation operation to induce chronic liver allograft dysfunction [12].

Group A (*n* = 36) included BN rat to Lewis rat transplantation with a 30-day course of low-dose subcutaneous Tacrolimus (0.1 mg/kg/day) treatment. Group B included control isogenic liver transplantations from BN rats to BN rats (*n* = 36) along with 30 days of low-dose subcutaneous Tacrolimus (0.1 mg/kg/day) treatment. In group C (*n* = 20), liver transplantation was performed from BN rats to Lewis rats with no immunosuppressive treatment. Each recipient rat was examined twice daily. For the survival experiment, recipient rats with CLAD were given either CXCL4mab (1 mg/kg) or physiological saline (PS) through the tail vein once every week from postoperative day (POD) 5 for 2 months. Antibodies against CXCL4 (sc-50300), CXCR3 (sc-9902), EGFR (sc-373746), JAK2 (sc-390539), STAT3 (sc-8019), Collagen IV (sc-167528), and GAPDH (sc-48166) were from Santa Cruz Biotechnology. Five recipient rats per group were allowed to survive until they died. Other recipient rats were killed on postoperation day (POD) 60. Blood samples were harvested from the inferior vena cava. The liver allografts were harvested for Masson staining, immunohistochemistry, Western blotting, and hepatic stellate cells (HSC) isolation.

### 2.3. Serum Biochemistry

Serum biochemistry analysis included aspartate aminotransferase (AST) and total bilirubin (TBIL) assessed by standard spectrophotometric methods using a HITAC7170A automatic analyzer (Hitachi, Tokyo, Japan). Statistically significant differences between groups were determined by one-way ANOVA (^*∗∗*^ *P* < 0.01; ^*∗*^ *P* < 0.05).

### 2.4. Liver Histopathological Analysis

Paraffin-embedded liver tissue sections of recipient rats were stained with hematoxylin and eosin (H&E). Liver fibrosis was analyzed on POD 60. For determination of fibrosis content, liver tissue samples were analyzed by Masson stain. Liver histopathological analysis was performed by a transplant pathologist who was blinded to the treatment group assignment. Independent sample *t*-tests were used for statistical analyses with *P* < 0.05 defined as statistically significant.

### 2.5. Quantitative Proteomic Analysis

Extracted proteins were assayed using the BCA method [13] and digested according to the FASP method [14]. iTRAQ labeling was performed according to the manufacturer's instructions (Applied Biosystems). iTRAQ quantitative proteomic analysis was performed on triplicate samples of recipient rats liver allografts and on three independent liver allografts as described [13, 15]. After acquiring MS/MS data with Q-Exactive software (Thermo-Fisher Scientific), all data files were processed using the Proteome Discoverer 1.3 software (AB SCIEX, Foster City, CA 94404, USA) for peptide identification. The user-defined search parameters were Enzyme: Trypsin; Static Modification: Carboxyamidomethylation (57.021 Da); Dynamic Modification: M Oxidation (15.995 Da) and carbamidomethyl (C), iTRAQ8plex (N-term), and iTRAQ8plex (K); false discovery rate (FDR) of all peptide and protein identifications: <1%; Max Missed Cleavages: 2; precursor ion mass tolerance: ±15 ppm; fragment ion mass tolerance: ±20 mmu. The Paragon™ algorithm v4.0 was used to identify the relative protein concentrations. Significant differences between the experimental and control groups were determined by ANOVA followed by Tukey's multiple range test. A difference ≥1.50-fold was considered evidence of upregulation, whereas a difference of ≤0.67-fold was considered to indicate downregulation. Protein expression levels were considered statistically significant if the *P* value was <0.05. To explore the pathogenesis of CLAD, Gene Ontology (GO) analysis was conducted using the DAVID program against the nonredundant protein database. Ingenuity Pathway Analysis (IPA, Ingenuity Systems, http://www.ingenuity.com/) used to investigated the functional relationships of discriminated proteins. We used IPA and its accompanying interaction database to analyze the data in the context of molecular mechanisms and relate these molecular changes with organism physiology and pathophysiology and to identify the ranked enrichment networks and canonical pathways for discriminated proteins. Fisher's exact test was used to determine the probability that each biological function assigned to that data set was due to chance alone.

### 2.6. HSC Isolation

HSCs were isolated from the liver allografts by a modified method as previously described [16, 17]. Briefly, liver grafts were perfused through the superior vena cava with 0.044% collagenase (Sigma, St. Louis, MO) and 0.5% pronase (Roche, Indianapolis, IN). Suspensions of liberated HSCs were prepared by centrifugation on a double-layered (17%/11.5%) metrizamide solution (Sigma, St. Louis, MO). After centrifugation at 20,000 rpm for 15 min, the HSCs were harvested from the top of the upper layer. HSC preparations with greater than 90% purity and viability were routinely obtained using this procedure, as determined by ultraviolet-excited fluorescence microscopy and Trypan blue dye exclusion, respectively. Isolated HSCs were used for* in vitro* experiments.

### 2.7. Construction of Recombinant Lentiviral Vector

The cDNA sequence of CXCL4 was obtained from GenBank. The shRNA target sequence for CXCL4 was 5′-CGCTGAAGAATGGGAGCAAAACTCGAGTTTTG CTCCCATTCTTCAGCG-3′. A scrambled fragment (5′-AGCGACGGAGATCTT AGCTGTCTCGAGACAGCTAAGATCTCCGTCGCT-3′) was used as a negative control. DNA oligonucleotides to produce the plasmid-based shRNA were cloned into the GV118 vector using the* Hpa*I/*Xho*I restriction sites. The lentiviral expression vector (GV118) and packaging vectors (pGC-LV, pHelper1.0 and 2.0) were cotransfected into 293T cells with Lipofectamine 2000 according to the manufacturer's instructions. The supernatant was collected 48 h later, centrifuged (4000 ×g, 4°C, 10 min) to remove cell debris, filtered through 0.45-*μ*m cellulose acetate filters, and then concentrated again (4000 ×g, 4°C, 15 min). The lentiviral vectors expressed green fluorescence protein (GFP), which allowed for titering and measurement of their infection efficiency in transduced cells. Isolated HSCs were dispensed into 6-well plates at a density of 50,000 cells per well and transduced with shRNA-expressing lentivirus at a multiplicity of infection (MOI) of 75.

### 2.8. Immunohistochemistry

Immunohistochemistry was performed for CXCL4 protein expression in five sections from per graft after the samples were fixed in 10% neutral-buffered formalin embedded in paraffin. The liver sections were incubated with a 1 : 100 dilution of anti-rabbit monoclonal CXCL4 antibody (Abcam, ab129183). Positive cells were counted at 400x magnification. Ten random fields were observed in each of the liver portal tract areas.

### 2.9. Western Blot Analysis

Liver allograft samples were rapidly ground in liquid nitrogen, and the cultured HSCs were harvested. The tissue powder or the isolated HSC cells were reconstituted in ice-cold RIPA buffer with protease and phosphatase inhibitors. Supernatants were recovered after centrifugation and assayed for protein content using the BCA method (Beyotime Biotechnology, P0010). Protein samples of 50 mg per lane were separated by 8% SDS-polyacrylamide gel electrophoresis (SDS-PAGE) and transferred to polyvinylidene-difluoride (PVDF) membranes. The membranes were incubated in 5% skimmed milk for 2 h at 37°C and overnight at 4°C with primary antibodies (Santa Cruz) (rat anti-CXCR3, 1 : 200 dilution; EGFR, 1 : 200 dilution; JAK2, 1 : 200 dilution; STAT3, 1 : 200 dilution; Collagen IV, 1 : 200 dilution). GAPDH protein expression was used as a loading control. The membranes were exposed to the negative films to develop target bands after incubation with enhanced chemiluminescence (Santa Cruz, USA). The intensities of bands were quantitated by LabWorks 4.5 software (UVP, USA).

## 3. Results

### 3.1. CXCL4 Is Associated with CLAD in Liver Transplant Patients

**Figure 1:**
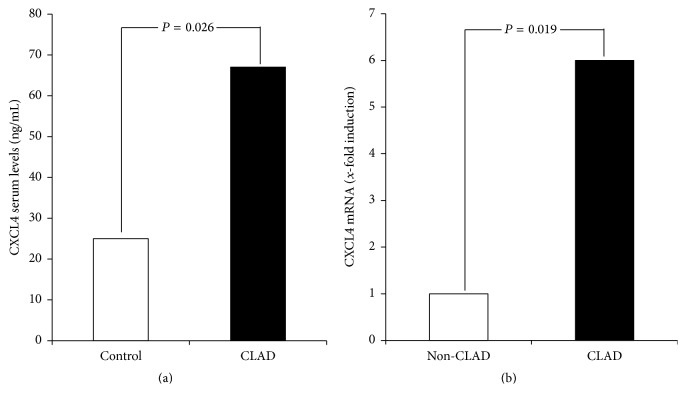
CXCL4 is associated with CLAD in liver transplant patients. (a) The serum concentration of CXCL4 was significantly increased in patients with CLAD (67.3 ± 3.1 ng/mL) compared to healthy controls (25.1 ± 2.6 ng/mL, *P* = 0.026). (b) CXCL4 mRNA was significantly increased in subjects with CLAD versus individuals without CLAD (*P* = 0.019).

We first evaluated an association of CXCL4 with CLAD in liver transplant patients. The serum concentration of CXCL4 was significantly increased in patients with CLAD compared to healthy controls (mean 66.70 ± 2.3 ng/mL, *P* = 0.026; Figure 1(a)).

We next validated whether hepatic CXCL4 mRNA expression was also increased in liver transplant recipients. Indeed, CXCL4 mRNA was significantly increased in subjects with CLAD versus individuals without CLAD (*P* = 0.019; Figure 1(b)). These results suggest that the upregulated CXCL4 is particularly associated with CLAD in liver transplant patients.

### 3.2. Histopathological Characteristics of Recipient Rat CLAD Liver

**Figure 2:**
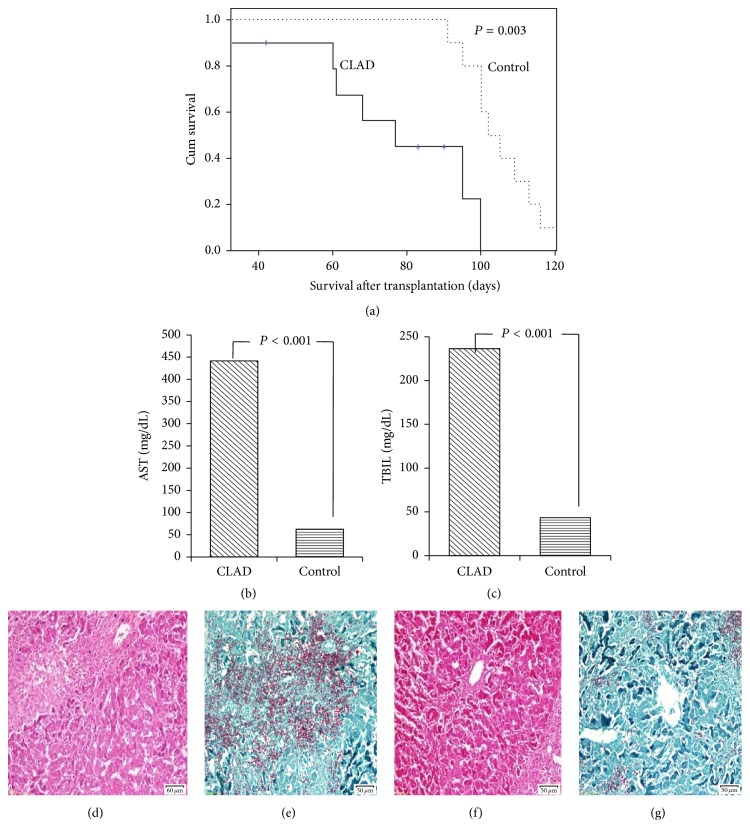
The characteristics of rat CLAD models. CLAD, allogeneic BN-to-Lewis rat liver transplantation with low-dose Tacrolimus Induction. Control, BN-to-BN rat liver transplantation with low-dose Tacrolimus Induction. (a) Recipient survival in different rat orthotopic liver transplantation models. The survival time was shorter in the CLAD group (62.8 ± 12.6 days) than in the control group (105.1 ± 19.3 days, *P* = 0.003). (b, c) Liver allograft function was evaluated by serum levels of AST and TBIL detected on POD 60. All CLAD liver grafts had moderate to severe dysfunction: AST 441.6 ± 101.6 mg/dL, TBIL 236.5 ± 91.6 mg/dL, but no apparent alterations were observed in Control group (AST 62.76 ± 15.6 mg/dL, *P* < 0.001; TBIL 43.3 ± 10.2 mg/dL, *P* < 0.001). (d–g) The pathologic characteristics of low-dose Tacrolimus-induced rat CLAD liver grafts at POD 60. HE stain ((d, e) ×200), Masson stain ((f, g) ×200). The pathology of the CLAD grafts was characterized by the loss of portal vein and loss of bile ducts in portal areas, intimal fibrous thickening without elastosis in hepatic arteries, and intraluminal fibrosis of the central veins with perivenular fibrosis, indicating the development of CLAD. No apparent histologic alterations were observed in the control group.

Low-dose Tacrolimus (0.1 mg/kg/day) treatment after liver transplantation was administered to recipient rats in the BN-to-Lewis transplantation group (group A). Survival in group A (recipient rats with CLAD) was 62.8 ± 12.6 days. This survival was greater in group B (the low-dose Tacrolimus-treated BN-to-BN group) (105.1 ± 19.3 days, *P* < 0.001) than in group A (Figures 2(a), 2(b), and 2(c)). However, all recipient rats in group C (BN-to-Lewis without Tacrolimus treatment group) developed irreversible acute rejection and succumbed to disease within 20 days. Liver allograft function was evaluated by determination of serum AST and TBIL levels. In group A, all liver grafts had moderate to severe dysfunction detected on POD 60 (AST 441.6 ± 101.6 mg/dL; TBIL 236.5 ± 91.6 mg/dL) but no apparent alterations were observed in the control group (group B) (AST 62.76 ± 15.6 mg/dL, *P* < 0.001; TBIL 43.3 ± 10.2 mg/dL, *P* < 0.001) (Figures 2(a), 2(b), and 2(c)). Manifestations of CLAD in this study, namely, obliterative arteriopathy, bile duct dystrophy or loss, and liver fibrosis, were consistent with the diagnostic criteria proposed for CLAD [18]. The architecture of the CLAD livers had changed from a lobular structure to a tissue largely composed of fibrotic nodules connecting portal tracts detected on POD 60. Obliterative arteriopathy and bile duct dystrophy or loss were more evident in CLAD liver allografts (Figures 2(a), 2(b), and 2(c)). However, no apparent histologic alterations and liver dysfunction were observed in the control group in which all rats survived >90 days.

### 3.3. Identifying CLAD-Associated Proteins Using iTRAQ Quantitative Proteomic Analysis

In this study, we investigated CLAD-associated proteins in liver allografts from the CLAD (BN-to-Lewis with Tacrolimus) and control groups (BN-to-BN with Tacrolimus). After validating the purity of the isolated proteins, quantitative proteomic analysis was performed. The proteins extracted from recipient rat livers were digested with trypsin. Biological groups containing proteins from the BN-to-BN group (113 or 115) and the BN-to-Lewis group were labeled with iTRAQ reagents (114 or 116) and then analyzed by nano-LC-MS/MS. Only one protein (sulfotransferase fragment) was identified without any iTRAQ labeling. Therefore, we set our acceptance criteria to include proteins with at least two unique labeled peptides. We then identified a total of 225 rat liver CLAD-associated proteins after combining four replicates. Of these, 98 proteins were reported previously, indicating the accuracy of the LD purification and proteomics techniques.

**Figure 3:**
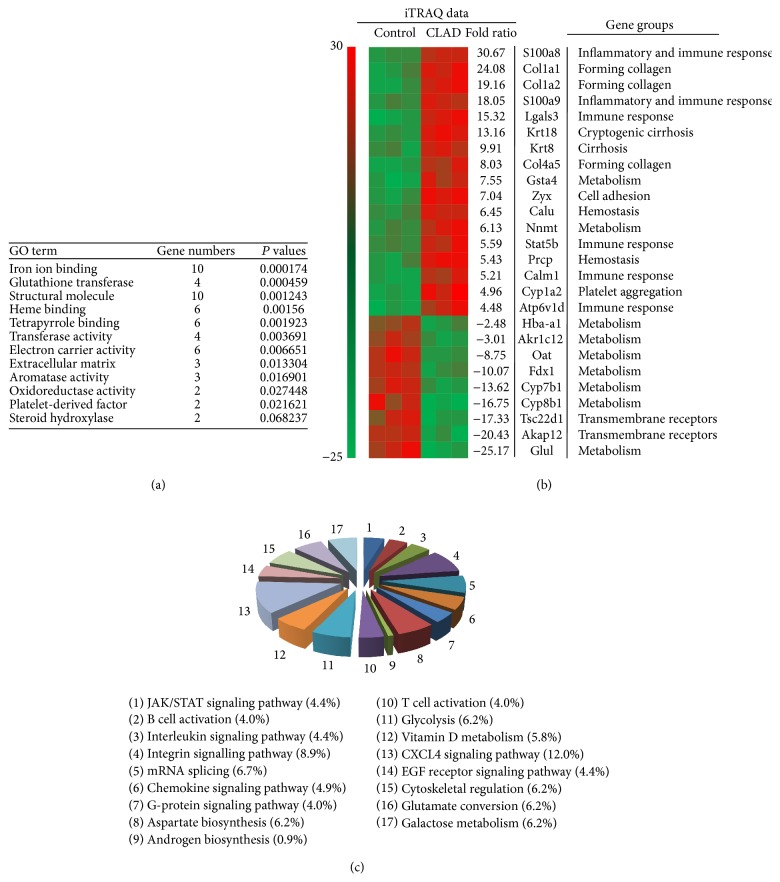
Quantitative proteomic analysis CLAD liver allografts. iTRAQ quantitative proteomics analysis was performed using protein samples from BN-to-BN liver allografts (control) and BN-to-Lewis liver allografts (CLAD). The proteins were analyzed with 95% or greater confidence as determined by ProteinPilot Unused scores (≤1.3). The following parameters were applied: an unpaired *t*-test with *P* value < 0.05, a false discovery rate (FDR) of all peptide and protein identifications <1%, and max missed cleavages and an enrichment score = 2. There was a ≥1.50-fold change for upregulation or ≤0.67-fold change for downregulation. (a) GO term analyses were performed using the online tool from DAVID Bioinformatics Resources (version 6.7). Both gene numbers and *P* values of specific gene groups were listed. (b) The top ranked 26 CLAD-associated proteins identified by functional annotation clustering/iTRAQ quantitative proteomic analysis. (c) The identified CLAD-associated proteins of liver allografts were categorized by subcellular distributions and functions based on PANTHER and Uniprot KB sources.

All identified proteins (225) were categorized into 17 groups according to their function and their assigned subcellular locations based on the PATRHE and GO TERM websites (Figures 3(a), 3(b), and 3(c)). The most unexpected findings were the identification of platelet-derived chemokines, epidermal growth factor receptor (EGFR), and the JAK/STAT signaling pathway in the pathogenesis of CLAD (Figures 3(a), 3(b), and 3(c)). Examining these groups, the platelet-derived chemokine CXCL4, JAK/STAT, and EGFR signaling pathways were significantly represented (20.9%, 47 proteins). Another major protein group was involved in the integrin signaling pathway (approximately 8.9%, 20 proteins), including several bidirectional signal transmitters for focal adhesion formation and fibroblast activation. This finding raised the possibility that CXCL4 expression, fibroblast activation, and fibrogenesis may be involved in CLAD. Cytoskeletal regulation by Rho GTPase (14, 6.2%), glutamine glutamate conversion (14, 6.2%), fructose galactose metabolism (14, 6.2%), Vitamin D metabolism (13, 5.8%), asparagine and aspartate biosynthesis (14, 6.2%), and glycolysis (14, 6.2%) were all notable liver metabolism proteins identified from CLAD recipient rat livers. Another notable group was composed of 18 proteins (approximately 8% of the total identified proteins) including the signaling proteins implicated in T cell and B cell activation. We also identified other cell signaling proteins including interleukin signaling (10, 4.4%), G-protein signaling (9, 4.0%), and androgen/estrogen/progesterone biosynthesis (2, 0.9%) in this study. Finally, there were a substantial number of mRNA splicing proteins (15; 6.7% of the total) not belonging to the other 16 categories, which indicated that some functions of CLAD-associated proteins are still unknown and will require further study.

### 3.4. Validating CLAD-Associated Proteins in Recipient Rat Liver Allografts

**Figure 4:**
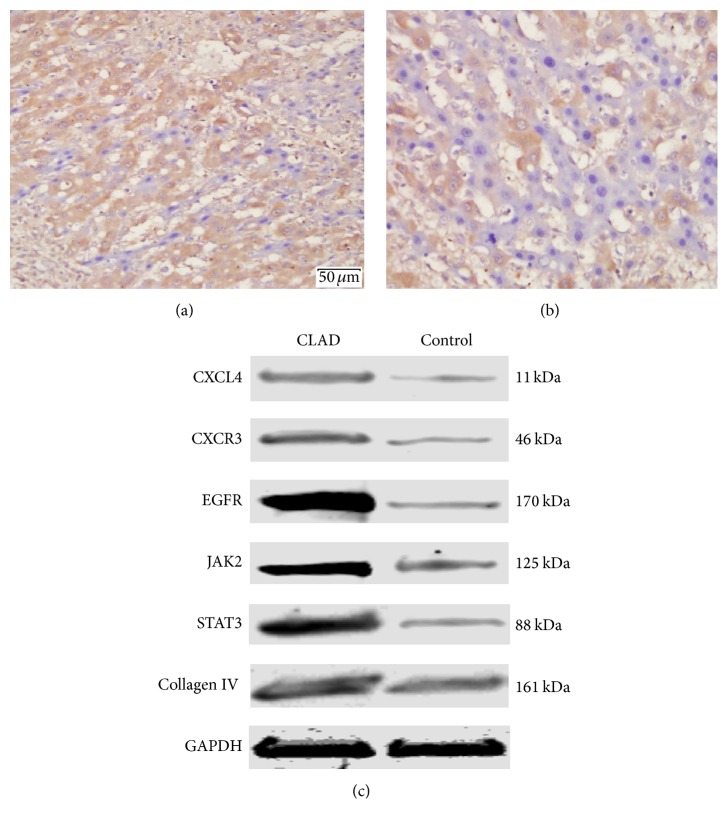
Validating CLAD-associated proteins in recipient rat liver allografts. (a) Immunohistochemistry analysis showed that CXCL4 detected on POD 60 was significantly expressed in CLAD liver allografts, compared with the control. (b) Validation by Western blot analysis on POD 60 showed that CXCL4, CXCR3, EGFR, JAK2, STAT3, and Collagen IV were significantly overrepresented in all CLAD liver grafts as compared with the control.

By pathway and biological function analysis, six differential proteins involved in CLAD or liver graft fibrosis, including CXCL4, CXCR3, EGFR, JAK2, STAT3, and Collagen IV, were selected for validation. Immunohistochemistry analysis showed that significantly greater expression of CXCL4 at POD 60 was observed in all liver allografts in the CLAD group (BN-to-Lewis) compared with those of the control group (BN-to-BN) (shown in Figure 4). When validated by Western blot analysis at POD 60, all six proteins were significantly overrepresented in all liver grafts in the CLAD group, compared with the BN-to-BN control group (shown in Figure 4). Taken together, these findings indicated CXCL4 and its receptor CXCR3, the EGFR signaling pathway, the JAK2/STAT3 signaling pathway, and collagen proteins participate in the pathogenesis of CLAD. These signaling pathways have potential therapeutic roles for liver fibrosis induced by HSC activation and CLAD after liver transplantation.

### 3.5. CXCL4 shRNA Reduces the Expression of Fibrogenesis-Associated Proteins in Isolated Hepatic Stellate Cells (HSCs)

**Figure 5:**
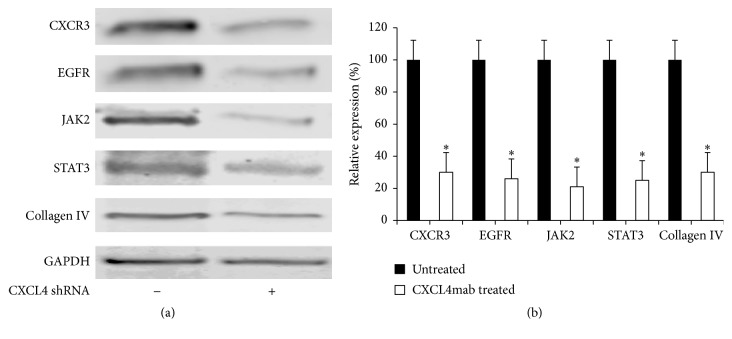
CXCL4 shRNA reduces the expression of fibrogenesis-associated proteins in isolated HSCs. (a) The isolated HSCs were transfected with CXCL4 shRNA for 72 hours. Western blot analysis of HSCs with antibodies against CXCR3, EGFR, JAK2, STAT3, and Collagen IV. (b) Quantitative presentation of these fibrogenesis-associated proteins. The CXCR3, EGFR, JAK2, STAT3, and Collagen IV levels in the CXCL4 shRNA-treated group decreased as a percent of their respective controls (untreated group). The data are presented as the mean ± SD, *n* = 3 liver grafts per group. ^*∗*^ *P* < 0.05 versus control group.

The activation of HSCs is a key event in the pathogenesis of CLAD. To understand the molecular mechanisms underlying the induction of CXCL4 expression in CLAD, the isolated HSCs were transfected with CXCL4 shRNA for 72 hours and examined for fibrogenesis by Western blot. CXCL4 shRNA-transfected HSCs were compared with untransfected HSCs. In this study, we found that CXCL4 shRNA significantly reduced CXCR3, EGFR, JAK2, STAT3, and Collagen IV expression in the transfected HSCs, but not in the control HSCs (shown in Figure 5) (*P* < 0.01). These data suggested that activation of HSCs is at least partially due to the induction of CXCL4. This result also suggests that CXCL4 is upstream of CXCR3, EGFR, JAK2, and STAT3 in the HSC activation pathways affected by CLAD.

### 3.6. Blockade of CXCL4 Protects Recipient Rat Livers from CLAD

**Figure 6:**
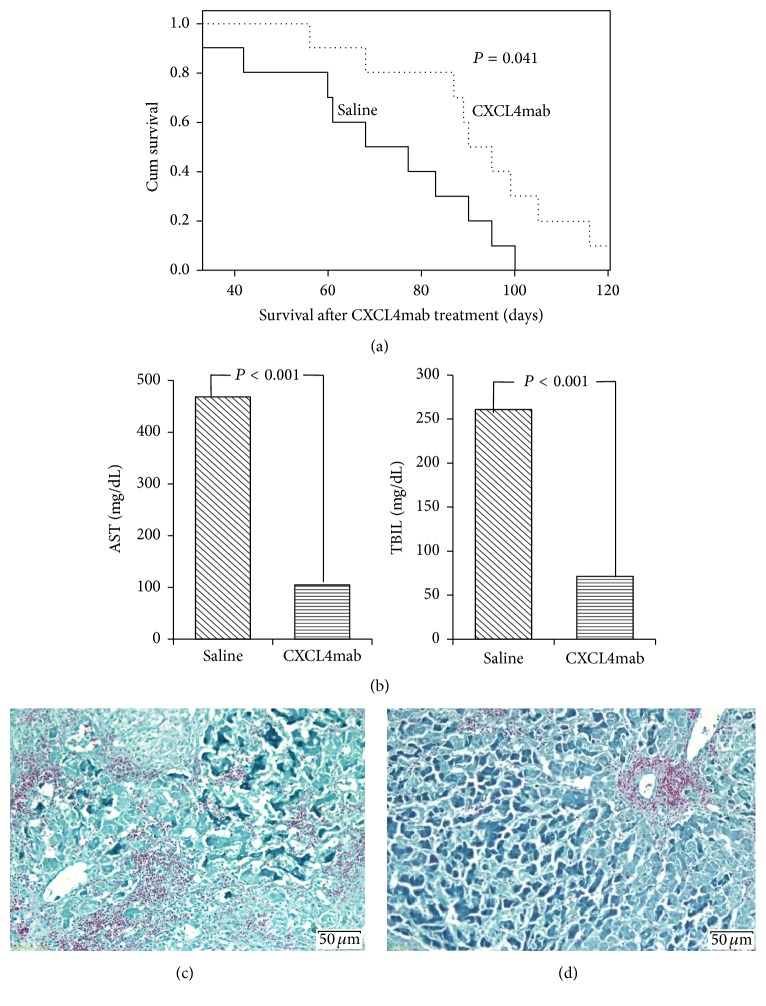
Blockade of CXCL4 protects recipient rat livers from CLAD. For the blockade experiment, either CXCL4mab (1 mg/kg) or physiological saline (control) was delivered to recipient rats (BN-to-Lewis) through the tail vein once every week from POD 5 for 2 months. (a) Survival time was more prolonged among recipient rats in the CXCL4mab-treated group than in the physiological saline group (*P* < 0.001). (b) Liver allograft function was evaluated by serum levels of AST and TBIL detected on POD 60. The AST levels in the CXCL4mab-treated group were significantly lower (105.1 ± 20.3 mg/dL) than those in the physiological saline group (468.6 ± 133.5 mg/dL, *P* < 0.001). A similar trend was observed for TBIL levels between the CXCL4mab-treated group (71.5 ± 19.7 mg/dL) and the physiological saline group (260.7 ± 84.6 mg/dL, *P* < 0.001). (c, d) Liver fibrosis of CLAD graft recipient rats was detected on POD 60 by Masson stain. CXCL4mab significantly alleviates recipient rat liver fibrosis after transplantation when compared with those in the physiological saline group.

Considering the important role of CXCL4 in recipient rat livers after transplantation, low-dose Tacrolimus-induced BN-to-Lewis rat CLAD models were established in this study. We used a neutralizing monoclonal antibody (CXCL4mAb) to block CXCL4 and to evaluate its protective roles in the pathogenesis of CLAD. For the blockade experiment, either CXCL4mab (1 mg/kg) or physiological saline were delivered to recipient rats (BN-to-Lewis) through the tail vein once every week for 2 months beginning on POD 5. The protective effect of the antibody was observed through serum biochemistry analysis, survival time after transplantation, and liver histopathological analysis. Importantly, CXCL4mab treatment alleviated recipient rat liver dysfunction after transplantation when compared with the physiological saline group (shown in Figure 6). Survival time was more prolonged among recipient rats in the CXCL4mab-treated group than in the physiological saline group (*P* < 0.001) (shown in Figure 6(a)). The AST levels in the CXCL4mab-treated group were significantly lower (105.1 ± 20.3 mg/dL) than in the physiological saline group (468.6 ± 133.5 mg/dL, *P* < 0.001). Similar trends were observed regarding TBIL levels between the CXCL4mab-treated group (71.5 ± 19.7 mg/dL) and the physiological saline group (260.7 ± 84.6 mg/dL, *P* < 0.001) (shown in Figure 6(b)). Liver fibrosis in the recipient rat CLAD grafts was detected at POD 60 by Masson stain (showed in Figures 6(c) and 6(d)). We found that CXCL4mab alleviates recipient rat liver fibrosis after transplantation when compared with rats in the physiological saline group.

## 4. Discussion

Previous studies have demonstrated that CXCL4 is involved in hematopoiesis, angiostasis, organ fibrogenesis, mitogenesis, tumor growth, and metastasis [1]. However, its role in the pathogenesis of CLAD has not been elucidated. In our study, to better understand CLAD, we analyzed rat liver transplantation samples with iTRAQ proteomics analysis and nano-LC-MS/MS. A total of 225 proteins were identified. These proteins were categorized into 17 groups according to their function and their assigned subcellular locations based on PATRHE and GO TERM analysis. We first identified CXCL4, CXCR3, EGFR, JAK2, STAT3, and Collagen IV as associated with CLAD pathogenesis and validated the relationship between CXCL4 and liver fibrogenesis in isolated HSCs. Indeed, liver graft fibrosis, a hallmark of CLAD, is a major cause of morbidity and mortality after liver transplantation [2].

The progression of CLAD was accompanied by liver graft fibrosis. We found that the protein expression of CXCL4 and its receptor CXCR3 were both upregulated in CLAD liver grafts. CXCL4 is secreted from platelets that specifically activate the CXCR3 receptor, which forms the basis for their pertinent involvement in the pathogenesis of CLAD [19]. A previous study showed that CXCL4 promotes fibrosis by platelet activation and aggregation, which activates or attracts hepatic stellate cells [20]. CXCL4(−/−) mice showed a significant reduction in histological and biochemical liver damage, which was accompanied by changes in the expression of fibrosis-related genes (TGF-beta [transforming growth factor beta], Mmp9 [matrix metalloproteinase 9], and Timp-1 [tissue inhibitor of matrix metalloproteinase 1]) [21]. More interesting, our findings also showed that CLAD was significantly ameliorated after blockade of CXCL4 in rat models (Figures 6(a)–6(d)).

The contribution of the CXCL4-CXCR3 axis to liver fibrosis has been delineated in recent years. CXCR3 is expressed on fibroblasts, epithelial and endothelial cells, smooth muscle, activated T lymphocytes, and dendritic cells. These cells express either CXCR3A, its splice variant CXCR3B, or a balanced combination of both [19]. CXCL4 activates both receptor variants during CLAD pathogenesis [1]. Stimulation of the CXCL4-CXCR3 axis leads to increased proliferation and activation of HSCs, which are not only a target but also a source of chemokines that contribute to the liver fibrogenesis [22]. To identify the signals downstream of CXCL4, CXCL4 shRNA was used to transfect HSCs isolated from CLAD liver grafts in this study. We found that CXCL4 shRNA significantly reduced the expression of downstream fibrosis-related proteins (CXCR3, EGFR, JAK2, STAT3, and Collagen IV) in HSCs (Figure 5).

EGFR, a transmembrane receptor tyrosine kinase, has been shown to play a key role during liver fibrosis following acute and chronic liver damage [23]. EGFR is expressed on HSCs, which are thought to be the major cell type involved in liver fibrosis [24]. Studies have shown that the EGFR inhibitor erlotinib prevented fibrosis progression by reducing EGFR phosphorylation in HSCs and lowering the total number of activated HSCs [24, 25]. Although these results suggest that EGFR inhibition might be a new therapeutic approach during liver fibrosis, the roles of EGFR and its ligands in CLAD after transplantation are not completely understood thus far.

Conditional deletion of CXCL4 (CXCL4 shRNA) significantly reduced expression of fibrogenesis-associated proteins (CXCR3, EGFR, JAK2, STAT3, and Collagen IV) in HSCs (Figure 5). These data suggest that CXCL4 is upstream of CXCR3, EGFR, JAK2, and STAT3 in HSC activation and liver graft fibrosis during CLAD. More interestingly, the significantly protective effects of CXCL4mab treatment in recipient rat liver dysfunction were observed in this study through serum biochemistry analysis, survival time after transplantation, and liver histopathological analysis. Taken together, these results indicated that the CXCL4-CXCR3 axis participates in the pathogenesis of CLAD and that CXCL4mab treatment is potentially beneficial in preventing and treatment of CLAD after liver transplantation.

In conclusion, our study provides the first dataset regarding CLAD liver allograft proteins and their variants under pathological conditions. CXCL4, CXCR3, EGFR, JAK2, STAT3, and Collagen IV were first identified as participating in the pathogenesis of CLAD in this study. We validated that CXCL4 is upstream of these informative genes in the isolated HSCs. CXCL4 has been identified as a mediator of fibrosis in CLAD liver grafts. Blocking CXCL4 protects against the pathogenesis of CLAD by reducing liver fibrosis. Therefore, our results add new insight into the mechanisms of CLAD after liver transplantation. Therapeutic approaches that neutralize CXCL4, the newly identified target of fibrosis, may represent a novel strategy for preventing and treating CLAD after liver transplantation.
